# Are There Benefits to Observation Units in the Emergency Departments: A Narrative Review

**DOI:** 10.3390/jcm14124333

**Published:** 2025-06-18

**Authors:** Emmeline Leggett, Shirin Haan, Carolina Mendoza, Ali Pourmand, Sarah Sommerkamp, Rose Chasm, Jason Adler, Michael C. Bond, Quincy K. Tran

**Affiliations:** 1 School of Medicine, University of Maryland, Baltimore, MD 21201, USA; 2 Research Associate Program in Emergency Medicine and Critical Care, Department of Emergency Medicine, School of Medicine, University of Maryland, Baltimore, MD 21201, USA; shaan@terpmail.umd.edu (S.H.); qtran@som.umaryland.edu (Q.K.T.); 3 Department of Emergency Medicine, School of Medicine, University of Maryland, Baltimore, MD 21201, USA; cmendoza@som.umaryland.edu (C.M.); ssommerkamp@som.umaryland.edu (S.S.); rchasm@som.umaryland.edu (R.C.); jason.adler@som.umaryland.edu (J.A.); michael.bond@som.umaryland.edu (M.C.B.); 4 Department of Emergency Medicine, School of Medicine and Health Sciences, George Washington University, Washington, DC 20052, USA; pourmand@gwu.edu; 5 Program in Trauma, The R Adam Cowley Shock Trauma Center, School of Medicine, University of Maryland, Baltimore, MD 21201, USA

**Keywords:** emergency department, observation units, ED length of stay, hospital admission

## Abstract

**Introduction:** Visits to Emergency Departments (ED) in the United States are increasing, creating a crowding problem, including longer length of stay in the ED (EDLOS) and worse outcomes. Many ED resort to observational units (EDOU) to help alleviate this crowding issue. This narrative review assessed the current state of literature to investigate the benefits of EDOU while reviewing the barriers to create such units. **Methods**: This review utilized the Patient Intervention Control Outcome (PICO) format. The searches were performed on PubMed from its inception to 14 November 2024. The outcomes were EDLOS, hospital admission rates, and 7-day ED return rates. Any randomized trials or observational studies (either retrospective or prospective) that reported pre-EDOUs and post-EDOUs, or studies comparing patients in the EDOUs versus control were eligible. We excluded abstracts and non-original studies. **Results**: Our search identified 904 results, and we included 34 articles in this review. Four studies reported EDLOS with an average of 14–23 h. Two studies performed a comparison analysis and found a decreases in EDLOS between 23 and 28%, while two studies discovered no significant difference. Four studies reported a statistically significant reduction in hospital admission rates, with absolute reductions in rates ranging from 2.7% to 44%. Two studies found no significant difference. Both EDLOS and rates of hospital admission were more impactful when EDOU focused on a single chief complaint or narrowed criteria. Only three studies commented on 7-day ED return rate, reporting ranges from 1.9% of patients returning in 72 h, and 10.8% returning within 14 days. Additionally, they identified that 53.3% of potentially avoidable visits occurred within 48 h of discharge, and the majority of returns were related to original chief complaints. **Conclusions**: The Observation Units for Emergency Departments offer many benefits such as potential reduction in EDLOS and hospital admissions. However, the implementation of EDOUs usually comes with high initial costs, which hinders the process. Thus, more studies with robust methodology are still needed to assess the intricacies of these benefits of the EDOUs.

## 1. Introduction

There has been an increasing number of visits among adults to the emergency departments (EDs) in the United States [1,2]. It was estimated that there were 43 ED visits per 100 people in 2021 [1]. This increasing trend caused significant crowding problems for both the EDs and their associated hospitals across the United States. As a result, EDs face numerous barriers to providing timely and effective care to their patients. A primary challenge is overcrowding—a multi-faceted consequence of inadequacies in hospital resources, including but not limited to space, staffing, and availability diagnostic testing [3]. When patients are slated for admission to the hospital, they remain in the ED due to lack of inpatient capacity; this further burdens and depletes the ED’s resources [3,4]. Boarding and overcrowding have unfavorable impacts on both patient care, outcomes, and the hospital’s costs and infrastructure [5,6]. A commonly proposed and implemented solution is Emergency Department Observation Units (EDOUs) [7].

Emergency Department Observation Unit (EDOUs) are a growing phenomenon, defined by the Society of Academic Medicine (SAEM) as units “dedicated to patient care areas for ED patients who require more care, but either do not meet admission criteria or are expected to be discharged within 24 h.” SAEM endorses using these units as a potential solution to reducing ED crowding, hospital diversion hours, and length of stay, but also notes that there is large variety in their current implementations [8].

One of the main reasons for the EDOUs is to avoid admitting patients to the hospital or to prematurely discharging certain patients from the ED. Patients with certain disease states or clinical characteristics remain in the ED to receive reassessment and diagnostic or therapeutic interventions [9]. These units can be diverse and accommodate a wide range of patients and disease states [10,11], and physicians or non-physician practitioners can manage them (NPPs) [12]. There are different benefits from the EDOUs, ranging from intangible, reducing ambulance diversion [13] to tangible, reducing costs for patients or hospitals [14].

Recent reviews about EDOUs have either been a broad overview about EDOUs [12] or they reviewed about the process of implementation [9]. For example, the recent review by Goodwin et al. examined the diagnoses for patients in the EDOUs, or the types of outcomes that were reported by studies involving EDOUs [12]. In contrast to the previous literature, this narrative review addresses the data regarding important and specific outcome metrics for Emergency Medicine (ED length of stay, hospital admission, rates of ED returns within 7 days) and how they are changed with implementation of an EDOUs. This review also addresses the barriers to implementation of EDOUs, so that clinicians and administrators can have a more in-depth view about EDOUs.

Accordingly, this manuscript is a narrative synthesis to gain insight into the effectiveness and outcomes from the implementation of EDOUs, addressing specifically the questions of how does the implementation of EDOUs impact hospital admission rates, patient outcomes, and healthcare costs? What are the variations, current or potential, in the implementation of EDOUs? What are potential challenges in the implementation of EDOUs?

## 2. Methods

A PubMed and SCOPUS search was performed, using a combination of the keywords “Emergency Department” AND “Observation Unit” OR (EDOU) AND (outcomes OR impacts) from their inceptions to 14 November 2024.

**Figure 1:**
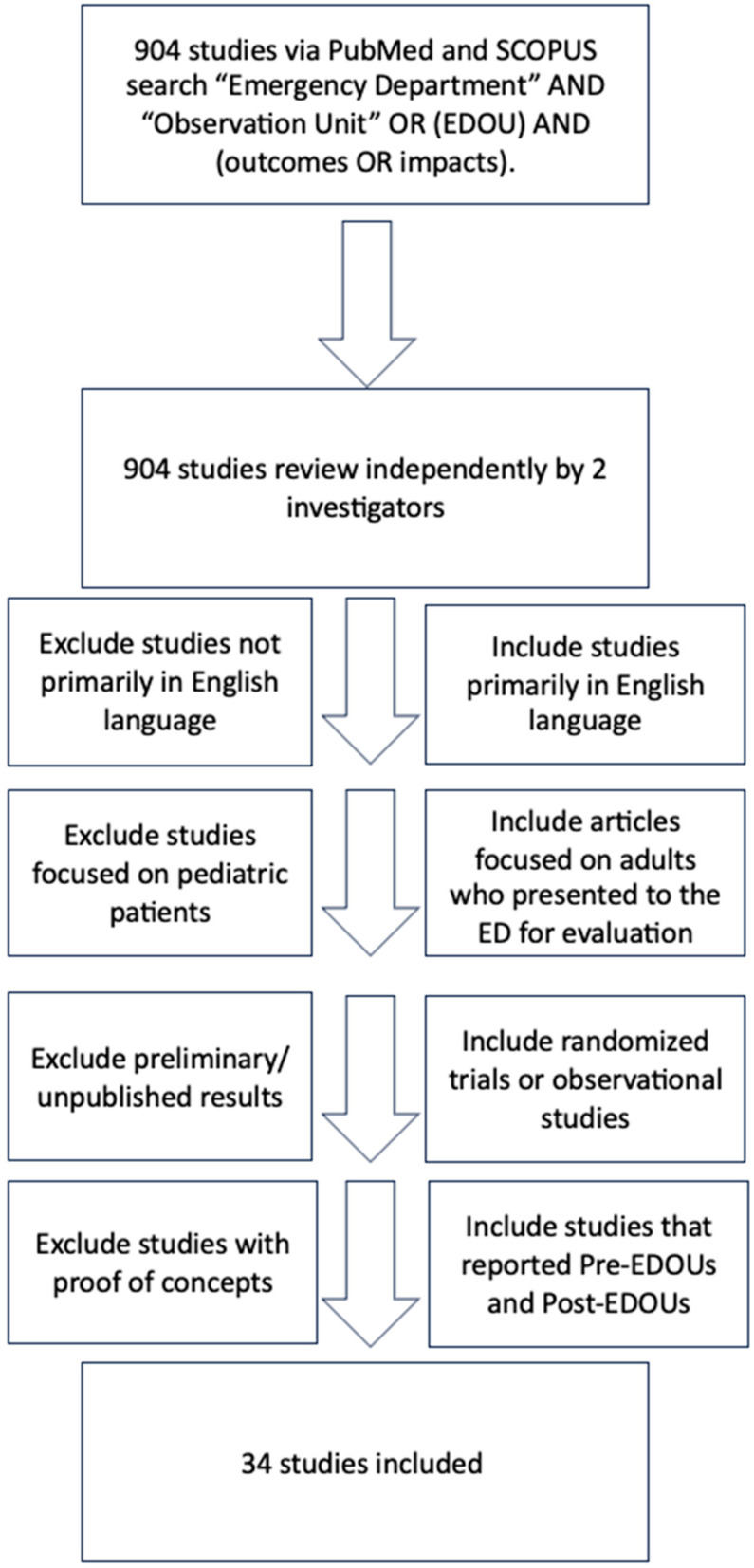
Flow diagram for the selection of studies.

Articles that present information regarding the observation units in the context of emergency medicine were included. The references of included studies were also reviewed to identify additional sources. Furthermore, additional references were added at the authors’ discretion. The initial literature search identified 904 articles, of which 34 articles were included in this review (Figure 1). This article is based on previously conducted studies and does not contain any studies with human participants or animals performed by any of the authors. Articles were not included if they were not primarily in English or did not have an English translation, were focused on pediatric patients, or were preliminary/unpublished results. Any randomized trials or observational studies (either retrospective or prospective) were eligible. Additionally, studies that reported pre-EDOUs and post-EDOUs or studies comparing patients in the EDOUs versus those not cared for by the EDOUs were eligible.

Our review utilized the Patient Intervention Control Outcome (PICO) format. The patient population was adult patients who presented to the ED for evaluation. Intervention was defined as the implementation of an EDOUs. The control population included those patients who were cared for in an ED before the start of the EDOUs, or they were not being cared for by the EDOUs. The outcomes of interest were hospital admission rates, 7-day ED return rates, and overall healthcare costs. For this narrative review of the EDOU implementation, we excluded studies of proof of concepts, as we were only interested in concrete results of the EDOUs. We also excluded other non-original studies (narrative reviews, systematic reviews), abstracts, or conference proceedings, as they do not have all of the necessary data for our purposes.

The results from the search terms were reviewed independently by 2 investigators. Any study that met inclusion criteria would be eligible for inclusion in the full-text step. Since this is a narrative review, there was no qualitative or quantitative synthesis of any of the data.

## 3. Results

### 3.1. Emergency Department Length of Stay

Our review included four (4) studies that reported concrete numbers for emergency department length of stay (EDLOS) (Table 1 and Table 2). Among these four, the average length of stay (LOS) reported approximately 14–15 h for patients within a multi-disciplinary EDOU [15], 23 h for patients in a cancer-specific EDOU [16], and around 18 h for the sickle cell dedicated EDOU [15]. However, these studies do not provide a comparative LOS to determine whether these metrics are an improvement from the typical EDLOS for each hospital system. Perry et al. addressed this knowledge gap by concluding through their observational study that hospitals with dedicated EDOUs with defined protocols noted decreases in EDLOS 23 to 38% when compared to those without [17]. Ross et al. observed a similar trend, citing that a hospitalist-run EDOU was associated with a 35% decrease in observation LOS [18]. Schull et al. found less of an impactful difference, with their pilot clinical decision unit reducing LOS for low acuity patients by 0.14 h and for non-admitted patients by 0.11 h [19]. Finally, only one study in our narrative review reported EDLOS, and it found no significant difference. Cheng et al. [20] hypothesized that the data in reduction in EDLOS is skewed because they are often targeted to one complaint, as exemplified above by the sickle cell EDOU or cancer EDOU. Thus, they evaluated two hospitals’ EDs both pre-EDOU implementation and post-implementation, both of which addressed a variety of complaints. They found that at ED A, there was an increase in the median ED LOS (179.0 min pre vs. 192.0 min post [+13.0 min]; *p* < 0.001), and no change in ED B (182.0 min pre vs. 182.0 min post) [20]. Ultimately, this data indicates that EDOUs with a narrowed focus on a single chief complaint can significantly decrease LOS, yet when they address a broader patient population, they have the potentially opposite effect of increasing LOS.

### 3.2. Rates of Hospital Admission

We identified seven studies that directly comment on the differences in the rates of hospital admissions with the implementation of EDOUs; however, there exists much variability in their conclusions. For instance, of these studies, four of them describe a reduction in hospital admissions. Schull et al. found a small, absolute hospital admission rate for all high-acuity patients (−0.8%, 95% CI = −1.5% to −0.03%) and moderate-acuity patients (−0.6%, 95% CI = −1.1% to −0.2%) [19]. Similarly, Brillman et al. identified a small, not statistically significant difference, finding only a 2.7% difference (*p* = 0.25). However, 5.3% fewer patients were admitted directly to the hospital (*p* = 0.01), and 6.7% fewer patients were discharged directly from the ED (*p* = 0.005) [23]. Two studies established considerably greater differences. Ross et al.’s study focused on EDOUs and the elderly, and they found that while elderly patients were more likely to be admitted from the EDOU than younger patients (26.1% versus 18.5%), their overall admission rate remained less than 30%, indicating a stark contrast to their hospital admission prior to EDOUs [18]. Ross et al.’s results mirrored those of the reduction in LOS, demonstrating that patients cared for in their “type 1” observation units had a 17–44 percent lower probability of subsequent inpatient admission [18]. In contrast, the remaining two studies found no significant difference in these metrics [17]. Similarly to the association of EDLOS, an essential factor in the success rates of decreasing admissions seems to be narrowed admission criteria for the unit. For instance, the observation unit pathway for overdose patients resulted in 88% of patients being discharged home without any reported deaths or need for airway management [42]. While this example differs in that it did not provide a direct comparison in admission rates, it exemplifies the idea that specific criteria and protocols are the cornerstone of a successfully implemented EDOU.

### 3.3. Returning to the ED Within 7 Days

There exists minimal information in the literature focusing specifically on the 7-day readmission rates to the ED, with only three studies identifying quantifiable metrics. Ross et al. identified that 10.8% of discharged patients returned within 14 days, with 7.9% relating to the initial visit. They found most return visits occurred within the first week, averaging 4.5 days [45]. Chaftari et al. looked specifically at cancer patients in EDOUs, and they found that they were successful in managing cancer-related pain with a 72 h ED return rate for unscheduled visits at a low 1.9% [44]. Berger et al. investigated the characteristics of patients who return to the ED after admittance to EDOU. They identified that the overall return rate from the EDOU was under 10%, with about two-thirds of the returns being related to the index visit. Importantly, they determined that 53.3% of potentially avoidable visits occurred within 48 h of discharge, suggesting this period as a potential quality metric for EDOU care. Additionally, they noticed that patients treated for asthma and chest pain had lower return rates, whereas patients treated for asthma had higher return rates [47]. Given the limited data on this metric and its immense relevance to the efficiency of EDs, this area is worth exploring further. One possible way to explore this topic is to consider both the patients’ characterization and the demographic of who is running the EDOUs. For instance, Mueller et al.’s study delved into three-day return rates in hospitalist versus ED physician-managed EDOUs for sickle cell patients, yet they found no statistical difference [21].

## 4. Discussion

### 4.1. Reduction in Costs

One area pertinent to the EDOU implementation conversation is their cost saving potential. The literature explained conflicting data and rationalizations on this topic. A majority of the researchers alluded to a significant reduction in costs for the hospital by referencing theoretical cost-saving elements of EDOUs such as decreasing unnecessary admissions, enhancing hospital bed capacity, and lowering the physician threshold for extended evaluation [33,40,42]. However, only two studies in our review offered quantifiable numbers demonstrating significant reduction. Most notably, one study identified an annualized savings of 10,399 bed days and USD 1,329,443 in total direct cost per year [23]. Likewise, a different study estimated USD 950 million in potential national cost savings each year [25].

Despite these promising numbers, it is important to note the start-up and indirect resource costs in implementing EDOUs. This cost obstacle is two-fold, involving both the hospitals’ financial distributions and insurer reimbursement’s ever-changing policies. As for the former, there are high upfront costs, and the potential savings once the EDOU is successfully implemented may never outweigh the initial financial and resource burden [18]. One must consider the space, physical resources, and staffing needed to implement a unit as complex as an EDOU. For the latter, securing consistent reimbursement for EDOU services from insurers is labor-intensive and challenging, and thus might shift costs from hospitals to patients [33]. Likewise, policy changes such as the “two midnight rule” in Medicare posit additional questions on successful implementation [19]. Accordingly, the question of cost is multifactorial and requires further probing. Based on the previous findings, it is evident that the EDOUs benefit, across all metrics, with a narrowed patient base and strict protocols. It would therefore be interesting to compare cost reduction metrics in broad spectrum EDOUs versus those that are narrowly focused to see if they follow similar trends.

### 4.2. Potential Challenges in the Implementation of EDOUs

Aside from start-up costs, a separate impediment to both the implementation and the research of EDOUs is their multitude of variations. We identified two broader organizational categories in the literature. The first, as mentioned previously, is implementation based on specific patient populations. For instance, many studies indicated that the narrower the patient focus, the more successful the EDOU, as it was easier to define protocols, hire appropriate staff, and streamline clinical decision-making. Some examples of notably successful units were those dedicated to acute psychiatric care, substance use disorder, and suspected acute coronary syndrome or chest pain [28,39,46]. The second category is implementation based on protocols or broader diagnoses groups. These included units dedicated to broader issues such as transition services or units defined by the staff such as hospitalist-run versus ED-run EDOUs [29,37]. Regardless of the defining characteristics, the overwhelming consensus in the literature was that the units must have effectively implemented, defined protocols, ideally including clear admission criteria, adequate and dedicated staffing, time limits, and clinical pathways [26,29].

While this variety can be positive, as hospitals can tailor EDOUs to their specific needs, the lack of set protocols leads to challenges in providing appropriate care. Henceforth, across all of the literature, researchers noted difficulties in adequately assessing objective metrics [17,18,25,31]. Without consistent benchmarks or measurements, there are significant gaps in knowledge about EDOUs and impediments to improvement in quality. While this narrative review attempts to synthesize the current literature in order to address these gaps, the conflicting findings and highly specific applications to each conclusion make it challenging to create any widely applicable conclusions. As a result of heterogeneity among the studies, future research should focus on a standardized set of outcome measures, tangible metrics, and protocols to improve comparability.

### 4.3. Limitations

This study serves as foundational exploration into the implementation of EDOUs; however, due to the narrow inclusion criteria and broad nature of a narrative synthesis, there are limitations to address. Primarily, as this is a relatively new topic and there is concern regarding availability of literature for a systematic review and meta-analysis, the decision was made to pursue a narrative review instead. This approach allows the review to serve as a preliminary guide for further in-depth research. Given the novelty of this topic and the lack of systematic reviews, we aimed to use this narrative review as an introduction to the topic; however, without a systematic approach to analysis, the paper has limitations in comprehensiveness and carries a potential for bias. For instance, the discussion regarding cost analysis is incomplete, lacking mathematical analysis and focus on indirect costs and long-term financial sustainability. Additionally, without stratifying results between disease groups, we cannot make any definitive claims about an EDOU’s impact on our metrics. Lastly, given that this review excluded non-English studies and conference abstracts, there is likely publication bias and omission of important data.

### 4.4. Actionable Items for Future EDOU Research and Implementation

Given the above-listed limitations, there are substantial avenues for future research on this topic. Firstly, using this narrative synthesis as a guide, this research should be expanded upon in a meta-analysis format. The data should therefore be analyzed systematically, focusing specifically on two areas of interest: cost and stratification by disease process. This review identified, in a broad sense, that EDOUs should reduce costs and that their effectiveness is decided upon by the narrowness of their scope. Thus, future research should delve into the specifics of these two topics with tangible financial data and analysis. Along similar lines, we alluded to policy changes such as the “two midnight rule” serving as a potential barrier for implementation, but this policy is only one of a multitude that impact the reimbursement system and hospital policy regarding EDOUs in the United States. Accordingly, future researchers should explore the intricacies of policy in EDOU implementation, and how the ever-changing political landscape may further provide barriers. In sum, future research should pre-identify standardized protocols for evaluation to make more substantiative claims, as EDOUs themselves are heterogenous in nature and the research follows suit.

## 5. Conclusions

The Observation Units for Emergency Departments offer many benefits. When focusing on caring for narrow ranges of disease states, EDOU can reduce ED length of stay. Similarly, the EDOU also offers other benefits such as potential overall cost savings and reduction in hospital admissions. However, the implementation of EDOUs often involves high initial costs, which hinders their adoption. There is a lack of consensus about what metrics are important to assess EDOUs’ performance. Thus, more studies with robust methodology are still needed to assess the benefits of the EDOUs.

## Figures and Tables

**Table 1:** Summary of characteristics from included studies.

Author	Study Type	Primary Outcome(s)	Secondary Outcome(s)
Mueller et al., 2015 [21]	Non-RCT Observational Study	Hospitalists managed sickle cell disease patients in an EDOU at a significantly higher admittance rate than ED physicians, but no significant difference in three-day return rates was found [21].	The average EDOU length of stay (LOS) for a sickle cell disease patient during the ED management period was 17 h and 54 min, while the average LOS during hospitalist management was 18 h and 23 min. The 30-day return rates for patients who did not return at three days were not significantly different [21].
Venkatesh et al., 2011 [22]	Non-RCT Observational Study	Observation care in U.S. EDs increased from 0.60% to 1.87% of visits between 2001 and 2008. Chest pain was the most common condition evaluated. One-third of hospitals had dedicated observation units by 2008 [22].	
J. Brillman et al., 1994 [23]	Non-RCT Observational Study	Observation units (OUs) in EDs for asthmatic patients result in lower initial discharge rates but do not significantly reduce hospital admissions [23].	
Schull et al., 2012 [19]	Non-RCT Observational Study	Clinical decision units in emergency departments can slightly reduce ED length of stay, admission rates, and no increase in ED revisit rates [19].	Clinical decision units were associated with reducing the length of stay for low-acuity and non-admitted patients [19].
Blecker et al., 2016 [24]	Non-RCT Observational Study	Increased observation unit availability may result in decreased hospitalizations and decreased home discharges for chest pain patients [24].	None
R. Roberts et al., 2001 [25]	N/A	Observation medicine in EDs improves resource utilization and patient care, with flexibility and creative solutions being key to successful implementation [25].	
Mace et al., 2003 [26]	Non-RCT Observational Study	Those hospitals that had OUs had a higher overall ED census, higher rate of diversion of ambulances, and were more likely to be in metropolitan areas, but there was no relationship to payor mix or to ED hospital admission rate [26].	
Cheng et al., 2016 [20]	Non-RCT Observational Study	A multi-diagnosis OU can reduce hospital admission rates, but does not significantly decrease ED length of stay [20].	
Perry et al., 2021 [17]	Non-RCT Observational Study	Within an academic medical center, EDOUs were associated with improved resource utilization and reduced cost. This represents a significant opportunity for hospitals to improve efficiency and contain costs [17].	Observation patients managed in an EDOU within an academic health system experienced shorter lengths of stay [17].
Parwana et al., 2018 [27]	Non-RCT Observational Study	Acute psychiatric OUs reduce ED boarding and length of stay, supporting efficient allocation of scarce inpatient psychiatric beds [27].	
Southerland et al., 2019 [28]	Non-RCT Observational Study	An EDOU can effectively care for a wide variety of patients requiring multiple consultations, procedures, and care coordination while maintaining an acceptable length of stay and admission rate [28].	
Ross et al., 2013 [29]	Non-RCT Observational Study	Type 1 OUs in EDs lead to shorter stays, lower inpatient admission rates, and potential annual savings of USD 5.5–8.5 billion [29].	
Ross et al., 2003 [18]	Non-RCT Observational Study	Elderly patients in EDOUs have effective lengths of stay and hospital admission rates, with comparable return visit rates to younger patients [18].	
Magarey et al., 2023 [30]	Systematic Review	Psychiatric OUs may reduce ED wait times for patients with mental health presentations, but more research is needed to confirm this [30].	Based on limited, poor-quality evidence, ED units may reduce LOS for patients with crisis mental health presentations [30].
Iannone et al., 2009 [31]	Non-RCT Observational Study	Short OUs in EDs benefit from proper organization and standardized clinical pathways, reducing re-admissions and hospitalizations [31].	Patients admitted to the short OU (SOU) from the ED had a shorter length of stay. Within 3 months post-discharge from the SOU, rates of ED visits and hospitalizations declined, while SOU re-admissions remained unchanged [31].
Kelen et al., 2001 [13]	Non-RCT Observational Study	An ED-managed acute care unit can significantly impact ED overcrowding and ambulance diversion, with no need for it to be proximate to the ED [13].	
Aplin et al., 2014 [32]	Non-RCT Observational Study	Implementing a hospitalist-run geographic CDU significantly reduced observation stay length without increasing ED or hospital revisit rates [32].	None
Komindr et al., 2014 [33]	Non-RCT Observational Study	EDOUs can improve efficiency and patient satisfaction by reducing length of stay, increasing bed turnover, and increasing discharge rates across both US and Asian sites [33].	None
Capp et al., 2015 [34]	Non-RCT Observational Study	The presence of EDOUs did not show a statistically significant decrease in ED hospital admission rates [34].	
Graff et al., 1992 [35]	Discussion Article	OUs in EDs improve patient care and help manage common emergencies, while contributing to the healthcare crisis and reducing costs [35].	
Koehler et al., 2009 [36]	Randomized controlled pilot study	A targeted care bundle for high-risk elderly inpatients reduced unplanned acute healthcare utilization up to 30 days after discharge, but this effect dissipated by 60 days post-discharge [36].	
Nuckols et al., 2017 [37]	Epidemiological analysis	Total returns to the hospital are stable or rising, likely due to growth in observation and ED visits [37].	
Zuckerman et al., 2016 [38]	Interrupted time-series analysis.	Hospitals have reduced readmission rates due to financial penalties under the ACA, but observation-unit stays did not significantly contribute to the decrease in readmissions [38].	
McWilliams et al., 2016 [39]	Randomized controlled trial	An integrated practice unit, called transition services, may reduce 30-day readmission rates for high-risk hospitalized patients [39].	
Cline et al., 2018 [40]	Prospective Cohort	Sickle cell anemia patients’ healthcare utilization varies significantly, with one cohort having more hospital admissions and ED encounters, while the other cohort had more day hospital encounters and used a sickle cell disease observation VOC protocol [40].	
Navas et al., 2022 [15]	Cross-sectional analysis	EDOUs services for suspected acute coronary syndrome are underused, with over half of potentially observation-amenable admissions paid for by Medicare and Medicaid [15].	None
Hostetler et al., 2002 [41]	Observational Retrospective Review	EDOUs can be a valuable tool for assessing and treating patients with questionable admitting criteria, but are not a substitute for inpatient units [42].	
Southerland et al., 2018 [42]	Observational Retrospective Review	An EDOU is a feasible setting for multidisciplinary geriatric assessments, resulting in targeted interventions and shorter lengths of stay [43].	
Mahadevan et al., 2010 [43]	Comprehensive Review	Observation services in EDs can reduce hospitalization costs and increase patient discharge without needing hospitalization, benefiting selected patients with critical diagnostic syndromes and emergency conditions [43].	
Hahn et al., 2024 [16]	Retrospective Cohort Study	The study identifies factors contributing to patients’ return to the ED post-observation, emphasizing the need for improved follow-up and discharge planning to reduce these returns [16].	
Chaftari et al., 2021 [44]	Non-RCT Observational Study	Placing cancer patients in EDOUs is safe, reduces admissions, and conserves hospital resources without compromising care [44].	Placing discharged ED patients in the EDOU instead may potentially lead to fewer short-term revisits [44].
Ross et al., 2010 [45]	Non-RCT Observational Study	EDOUs recidivism rates differ by observation category, with painful conditions showing the highest recidivism rates [45].	
Shastry et al., 2020 [46]	Non-RCT Observational Study	OUs can safely manage low-risk drug overdose patients in EDs, with acceptable adverse event rates [46].	
Baerger et al., 2013 [47]	Non-RCT Observational Study	The overall return rate from EDOUs is under 10%, with two-thirds of returns related to the index visit and 5% potentially avoidable [47].	

**Table 2:** Summary of major findings from the included studies.

Category	Primary Findings	Main Takeaway	Limitation(s)	Future Direction(s)
EDLOS	Average EDLOS reported among four studies was between 14 and 23 h, dependent upon the specific disease process(es) treated. With defined protocols and narrowed disease focus, EDLOS was decreased by a statistically significant amount. EDOUs that addressed a variety complaints found no statistical difference in EDLOS with a mild increase.	EDOUs with a narrowed focus on a single chief complaint can significantly decrease LOS, yet when they address a broader patient population, they have the potentially opposite effect of increasing LOS.	Heterogeneity among EDOU protocols creates difficulty making direct comparisons. Some of the studies that report EDLOS did not provide a comparison number for their ED without EDOU implementation.	Meta-analysis of EDOUs stratified by disease process.
Rate of Hospital Admission	Four studies identified a significant reduction in hospital admission rates. Among these, the authors noted that narrowed admission criteria contributed to this difference. Two studies identified no significant difference in hospital admission.	A narrowed population or disease focus contributed positively to a reduction in hospital admission rates.	Heterogeneity among EDOU protocols creates difficulty making direct comparisons.	Meta-analysis of EDOUs stratified by disease process.
Return to ED in 7 Days	Three studies reported quantifiable metrics, finding low return rates and the majority of return rates were due to original complaint. 53.3% of potentially avoidable visits occurred within 48 h of discharge.	Return to ED in 7 days was not a significant finding in much of the literature, yet of those that reported metrics, they were low.	Limited data on specific focuses on 7-day readmission rates to the ED.	Meta-analysis of EDOUs stratification by disease process. Investigation into differences in ED return rates based on demographics of who runs EDOU.
Reduction in Costs	Potential reduction in costs by decreasing unnecessary admissions, enhancing bed capacity, lowering physician threshold for extended evaluation High start-up and indirect resource costs Difficulties in reimbursement policies	Majority of the researchers alluded to a significant reduction in costs for the hospital by referencing theoretical cost-saving elements of EDOUs such as decreasing unnecessary admissions, enhancing hospital bed capacity, and lowering the physician threshold for extended evaluation.	Only two studies in our review offered quantifiable numbers demonstrating significant reduction in costs. Economic analysis lacks depth Lack of in-depth investigation into reimbursement policies.	Meta-analysis of EDOUs focusing specifically on costs Further exploration into reimbursement and hospital policies.
Challenges in Implementation	Heterogeneity in implementation of EDOUs creates impediments to quality improvement and protocol development.	Heterogeneity in implementation of EDOUs creates impediments to quality improvement and protocol development.	Heterogeneity in implementation of EDOUs creates gaps in research availability and comparative data.	Meta-analysis of EDOUs to assess EDOUs in a more systematic manner.
